# Chikungunya-related Guillain-Barre syndrome is most commonly demyelinating and affects multiple cranial nerves

**DOI:** 10.1186/s42466-024-00336-1

**Published:** 2024-11-18

**Authors:** Josef Finsterer

**Affiliations:** Neurology Department, Neurology & Neurophysiology Center, Vienna, Austria

**Keywords:** AMSAN, Chikungunya, Nerve conduction studies, Cranial nerves, Guillain Barre syndrome

Letter to the editor,

We read with interest Sreelakshmi et al’s article about a 21-year-old female with Guillain-Barre syndrome (GBS), following infection with Chikungunya four weeks earlier [1]. Due to respiratory failure, the patient required intubation and mechanical ventilation for two weeks [1]. Despite immediate administration of immunoglobulins, the patient’s condition initially deteriorated and multiple cranial nerve involvement occurred: However, after a second immunoglobulin cycle, a steady and rapid improvement in most deficits was achieved [1]. The study is impressive, but several points require discussion.

The first point is that the patient was diagnosed with acute motor and sensory axonal neuropathy (AMSAN) although the results of nerve conduction studies (NCSs) were described as axonal and demyelinating [1]. AMSAN is characterised by axonal lesions in the NCSs but not by demyelinating lesions. This discrepancy should be resolved. Since patients with Chikungunya-related GBS most commonly manifest with acute inflammatory demyelinating polyneuropathy (AIDP), the demyelinating subtype of GBS [2], it is more likely that the index patient had AIDP with secondary axonal lesions than AMSAN. In a study of 13 patients with Chikungunya-related GBS during the 2014 outbreak in Martinique and Guadeloupe, 54% of these patients were diagnosed with AIDP, 15% with AMSAN, 15% with Miller-Fisher syndrome (MFS), 8% with the pharyngeal-cervico-brachial subtype and 8% with Bickerstaff encephalitis [2].

The second point is that the patient was intubated and mechanically ventilated due to muscular respiratory insufficiency, and subsequently underwent a clinical neurological examination, which revealed quadriparesis and paresthesias [1]. We should know how it was possible to diagnose muscle weakness in an intubated and relaxed patient under mechanical ventilation. This discrepancy should be resolved. It is also not understandable why paresthesia was found during the clinical neurological examination. Paresthesia is usually a symptom and is therefore reported by the patient. How could an intubated and ventilated patient report paresthesia? This discrepancy should be resolved.

The third point is that the cerebral MRI was reported as normal, but Bickerstaff encephalitis was still considered. What was the reason for this consideration? Was the MRI performed with contrast medium and was it possible to detect an enhancing lesion in the brainstem? Ophthalmoparesis, facial palsy, and dysphagia may not only be explained by Bickerstaff encephalitis, but could also be due to multiple cranial nerve involvement, another subtype of GBS. Involvement of the cranial nerves could be documented by MRI of the skull base with contrast showing enhancement of the cranial nerve roots [3].

The fourth point is that skeletal muscle impairment was not taken into account and therefore not excluded. Since Chikungunya can cause myositis [4], it would have been mandatory to measure creatine-kinase, lactate dehydrogenase, aldolase, and myoglobin, record needle electromyography of weak muscles, and determine myositis specific and myositis-associated antibodies.

The fifth point is that a SARS-CoV-2 infection could not be definitively ruled out as a trigger for GBS. Since the case obviously falls during the pandemic, it would have been mandatory to perform RT-PCR for SARS-CoV-2.

In summary, the excellent study has limitations that should be addressed before drawing final conclusions. Clarifying the weaknesses would strengthen the conclusions and could improve the study. Chikungunya-related Guillain-Barre syndrome is most commonly demyelinating and can affect multiple cranial nerves.

## Data Availability

All data are available from the corresponding author.
